# Circ-AFF2/miR-650/CNP axis promotes proliferation, inflammatory response, migration, and invasion of rheumatoid arthritis synovial fibroblasts

**DOI:** 10.1186/s13018-021-02306-8

**Published:** 2021-03-02

**Authors:** Wei Qu, Ling Jiang, Guanhua Hou

**Affiliations:** 1 Department of Joint and Sports Medicine, Weihai Municipal Hospital, Cheeloo College of Medicine, Shandong University, Weihai, 264200 Shandong China; 2 Department of Medical, Zibo Social Welfare Institute, Zibo, Shandong China; 3 Department of Orthopedics, Peking University Medical Zibo Hospital, No.2, 5Th Street, Shanlv Xishan, Nanding Town, Zhangdian District, Zibo, Shandong China

**Keywords:** Rheumatoid arthritis, Rheumatoid arthritis fibroblast-like synoviocytes, Circ-AFF2, MiR-650, CNP

## Abstract

**Background:**

Circular RNAs (circRNAs) are associated with rheumatoid arthritis (RA) development. The purpose of this study is to explore the function and mechanism of circRNA fragile mental retardation 2 (circ-AFF2) in the processes of rheumatoid arthritis fibroblast-like synoviocytes (RAFLSs).

**Methods:**

Circ-AFF2, microRNA (miR)-650, and 2′,3′-cyclic nucleotide 3′-phosphodiesterase (CNP) levels were determined in synovial tissues of RA and RAFLSs by quantitative reverse transcription polymerase chain reaction or Western blotting. Cell proliferation, inflammatory response, apoptosis, caspase3 activity, migration, invasion, and epithelial-mesenchymal transition (EMT) were investigated using Cell Counting Kit-8 (CCK-8), enzyme-linked immunosorbent assay (ELISA), flow cytometry, Transwell, and Western blotting analyses. Dual-luciferase reporter, RNA immunoprecipitation (RIP), and pull-down assays were performed to assess the binding relationship.

**Results:**

Circ-AFF2 expression level was enhanced in synovial tissues of RA and RAFLSs. Circ-AFF2 overexpression facilitated cell proliferation, inflammatory response, migration, invasion, and EMT and repressed apoptosis in RAFLSs. Circ-AFF2 downregulation played an opposite role. Circ-AFF2 targeted miR-650, and miR-650 downregulation reversed the effect of circ-AFF2 interference on RAFLS processes. CNP was targeted by miR-650, and circ-AFF2 increased CNP expression by regulating miR-650. MiR-650 overexpression constrained cell proliferation, inflammatory response, migration, invasion, and EMT and contributed to apoptosis by decreasing CNP in RAFLSs.

**Conclusion:**

Circ-AFF2 promoted proliferation, inflammatory response, migration, and invasion of RAFLSs by modulating the miR-650/CNP axis.

## Introduction

Rheumatoid arthritis (RA) is an autoimmune disease with common symptoms like musculoskeletal pain, swelling, and stiffness, which can impair physical function and life quality of patients [1]. The synovial tissue is a membranous organ lining joint cavities, which functions in joint destruction in RA [2]. Fibroblast-like synoviocytes are the dominant cells of synovial tissues, which contribute to cartilage destruction and RA development by interacting with immune cells and taking part in the inflammatory response of synovial joints [2, 3]. Moreover, fibroblast-like synoviocytes have important roles in the onset of RA [4]. Identification of fibroblast-like synoviocyte function in the present study fits into the framework of translational orthopedics by filling the gap between basic sciences and clinical sciences [5, 6]. Therefore, illustrating the mechanism of fibroblast-like synoviocyte processes might help to understand RA pathogenesis.

Noncoding RNAs are involved in the autoimmunity and inflammation that play important roles in RA development [7]. Circular RNAs (circRNAs) are a type of closed noncoding RNAs which bind with microRNA (miRNA) to reduce miRNA activity, subsequently increasing mRNA stability, which have key roles in aging-related diseases, including RA [8]. Moreover, the dysregulated circRNAs are associated with the function of fibroblast-like synoviocytes in RA [9]. For example, hsa_circ_0088036 increases fibroblast-like synoviocyte proliferation and migration by modulating miR-140-3p and Sirtuin (SIRT)-1 [10]. CircRNA fragile mental retardation 2 (circ-AFF2; hsa_circ_0001947) is an upregulated circRNA in the peripheral blood of RA patients [11]. However, the function of this circRNA and how it participates in fibroblast-like synoviocyte processes in RA are mostly uncertain.

MiRNAs are another type of noncoding RNAs that have important functions in musculoskeletal conditions, such as RA, osteoarthritis, and tendon injuries [12–14]. Furthermore, miRNAs are related to the function of fibroblast-like synoviocytes in RA [15]. A previous study suggests miR-650 represses proliferation, migration, and invasion of rheumatoid arthritis synovial fibroblasts [16]. However, whether miR-650 is relevant to the function of circ-AFF2 in fibroblast-like synoviocytes remains uncertain. 2′,3′-Cyclic nucleotide 3′-phosphodiesterase (CNP) can prevent cartilage damage in inflammatory arthritis [17]. Moreover, CNP level is enhanced in RA patients [18]. Yet no study has been done to analyze the function of CNP in fibroblast-like synoviocytes.

In this study, we analyzed the function of circ-AFF2 on fibroblast-like synoviocyte processes in RA and explored the network of circ-AFF2/miR-650/CNP. This study might provide a novel insight into the pathology of RA.

## Materials and methods

### Bioinformatic analysis

The circular structure of circ-AFF2 (hsa_circ_0001947) was analyzed using CircView (http://gb.whu.edu.cn/CircView/) [19]. The targets of circ-AFF2 were searched by starBase (http://starbase.sysu.edu.cn/) [20]. The targets of miR-650 were analyzed utilizing miRDB (http://mirdb.org/) [21], miRwalk (http://mirwalk.umm.uni-heidelberg.de/) [22], starBase, and Tarbase (http://carolina.imis.athena-innovation.gr/diana_tools/web/index.php?r=tarbasev8/index) [23].

### Patients and tissues

The synovial tissues of RA were obtained from RA patients (*n* = 34) who were diagnosed as RA and suffered from joint surgery at Weihai Municipal Hospital. All RA patients fulfilled the American College of Rheumatology criteria. The age- and gender-matched patients with joint trauma were regarded as the normal group, and the synovial tissues from the normal group (*n* = 23) were collected during the joint surgery. The patients with autoimmune or infectious disease were excluded from the normal group. All participants signed the written informed consent. This study complied with the guidelines of the Declaration of Helsinki and was authorized by the ethics committee of Weihai Municipal Hospital.

### RAFLS isolation and culture

The fibroblast-like synoviocytes were isolated from synovial tissues of RA (RAFLSs) or normal subjects (normal cells). Briefly, the synovial tissues were cut into pieces and subjected to 0.1% type-I collagenase (Solarbio, Beijing, China) digestion at 37 °C for 4 h. After that, cells were collected after centrifugation at 1000*g* for 5 min and cultured in Dulbecco’s modified Eagle’s medium (DMEM) (Gibco, Grand Island, NY, USA) plus 10% fetal bovine serum (FBS) (Gibco) and 1% penicillin/streptomycin (Gibco) at 37 °C with 5% CO_2_.

### Quantitative reverse transcription polymerase chain reaction (qRT-PCR)

RNA was extracted using Trizol (Vazyme, Nanjing, China) according to the instruction. The RNA from the nucleus or cytoplasm of RAFLSs was extracted using a cytoplasmic and nuclear RNA purification kit (Norgen Biotek, Thorold, Canada) according to the manufacturer’s instruction. RNA (800 ng) was applied for cDNA synthesis using a miRNA or M-MLV reverse transcriptase kit or (Thermo Fisher Scientific, Waltham, MA, USA). The cDNA was mixed with SYBR (Vazyme) and primer pairs (Sangon, Shanghai, China) and utilized for qRT-PCR. The specific primer sequences are shown in Table 1. Relative RNA expression was calculated using the 2^-ΔΔCt^ method, with U6 or GAPDH as an internal control.

**Table 1:** The primer sequences for qRT-PCR in this study

Name	Sequence (5′-3′)	
	Forward	Reverse
miR-3612	GCCGAGAGGAGGCATCTTGAG	AGTGCAGGGTCCGAGGTATT
miR-650	GCCGAGAGGAGGCAGCGCTC	AGTGCAGGGTCCGAGGTATT
U6	CAGGTCTCGGGAGAGAGATCG	TGTCGTCTTGGAGATCGGGAG
circ-AFF2	TCTTGGATGGAAAACCCAGT	AGTTTCCAAGCGTGTTCTGG
CNP	GGAAAGCGCACGCTTAGGAG	GGCAGGAATGTGTGGCTTTT
ANKRD52	TGGCGAGACCGGAATCCT	CCTTTTTCCAGGAGGGGTGG
AXL	CACGCGTAAACAACACGCA	GTTATGGGCTTCGCAGGAGA
HNRNPUL1	CTGCTTTCTGGAGCCGAAGA	GGGTGCTCCTTGCTTCATCT
APBB2	CTCCTTTGTTTGCAGGGATTTTGC	TGGCATTCTTCCGTTCAGCC
RAC1	TGATGCAGGCCATCAAGTGT	AGAACACATCTGTTTGCGGA
CTSB	ATCATGTGGGTGAGCCAGTG	TGGGGCAGCGAGAAGTTAAG
PLEC	TCATCCAGGCCTACGAGGAG	CCAGCAGGGAGATGAGGTTG
CD274	TTGCTGAACGCCCCATACAA	CCCCGATGAACCCCTAAACC
GAPDH	TTCTTTTGCGTCGCCAGGTG	GGAGGGAGAGAACAGTGAGC

### Cell transfection

Circ-AFF2 overexpression vector was constructed by Geneseed (Guangzhou, China), with pCD5-ciR vector as a negative control (vector). CNP overexpression vector was constructed in our laboratory, with pcDNA3.1 (Thermo Fisher Scientific, Waltham, MA, USA) as a negative control (pcDNA). Small interfering RNA (siRNA) for circ-AFF2 (si-circ-AFF2), siRNA negative control (si-NC), miR-3612 mimic, miR-650 mimic, mimic negative control (miR-NC), miR-650 inhibitor (anti-miR-650), and inhibitor negative control (anti-NC) were generated by Genomeditech (Shanghai, China), and the related sequences are displayed in Table 2. RAFLSs were transfected with 2 μg vectors or 40 nM oligonucleotides using Lipofectamine 3000 (Thermo Fisher Scientific) for 24 h according to the instruction.

**Table 2:** The oligo sequences for transfection in this study

Name	Sequence (5′-3′)
si-circ-AFF2	ACCUUUGUUUGUUUCACUUGU
si-NC	UGGAAAGAAACAUUCACUUGU
miR-3612 mimic	AGGAGGCAUCUUGAGAAAUGGA
miR-650 mimic	AGGAGGCAGCGCUCUCAGGAC
miR-NC	CGAUCGCAUCAGCAUCGAUUGC
anti-miR-650	GUCCUGAGAGCGCUGCCUCCU
anti-NC	CAGUACUUUUGUGUAGUACAA

### Cell Counting Kit-8 (CCK-8)

RAFLSs (1 × 10^4^/well) were dispersed in 96-well plates. After 0, 24, 48, or 72 h, 10 μL CCK-8 (Solarbio) was infused, and cells were continuously nurtured for 3 h. The optical density (OD) level at 450 nm was detected using a microplate reader (Bio-Rad, Hercules, CA, USA).

### Enzyme-linked immunosorbent assay (ELISA)

The secretion levels of pro-inflammatory interleukin (IL)-6 and anti-inflammatory IL-10 were detected by ELISA. In brief, 5 × 10^4^ RAFLSs were placed in 24-well plates and incubated for 48 h. The culture supernatants were collected and used for the detection of IL-6 and IL-10 secretion levels using an IL-6 or IL-10 ELISA kit (Thermo Fisher Scientific) following the instructions. The absorbance at 450 nm was detected with a microplate reader.

### Flow cytometry

An Annexin V-fluorescein isothiocyanate (FITC) apoptosis detection kit (Beyotime, Shanghai, China) was used for apoptosis detection according to the instruction. In brief, 2 × 10^5^ RAFLSs were dispersed in 12-well plates for 72 h. Then cells were collected, resuspended in Annexin V binding buffer, and stained with 10 μL Annexin V-FITC and propidium iodide (PI) for 10 min in the dark. A flow cytometer (Beckman Coulter, Brea, CA, USA) was used to detect the apoptotic rate.

### Caspase3 activity analysis

Caspase3 activity was analyzed using a caspase3 activity assay kit (Beyotime) following the instructions. In brief, RAFLSs were incubated for 72 h, and then 2 × 10^6^ cells were collected and lysed in 100 μL Caspase cell lysis buffer. Next, 50 μL lysates were placed into 96-well plates and incubated with 40 μL Caspase reaction buffer and 10 μL 2 mM Ac-DEVD-AFC at 37 °C for 120 min. The absorbance at 405 nm was examined through a microplate reader. Relative caspase3 activity was shown as the fold change of the control group.

### Transwell analysis

Transwell migration analysis was processed using 24-well Transwell chambers (Corning Inc., Corning, NY, USA) with a fibronectin-coated polycarbonate membrane. 1 × 10^5^ RAFLSs in serum-free DMEM were placed in the upper chambers, and 500 μL DMEM with 10% FBS was infused in the lower chambers. After 24 h, RAFLSs that passed the membrane were stained with 0.1% crystal violet (Solarbio) and imaged under a × 100 magnification microscope (Olympus, Tokyo, Japan). For Transwell invasion analysis, the membranes were pre-coated by Matrigel (Solarbio), and 5 × 10^5^ RAFLSs in non-serum DMEM were dispersed in the upper chambers. The other procedures were the same with the Transwell migration analysis. The migratory or invasive cell numbers were analyzed by Image J (NIH, Bethesda, MD, USA).

### Western blotting

After isolating in radioimmunoprecipitation assay buffer (Solarbio), protein was collected by centrifugation at 10,000×*g* for 10 min, and the concentration was detected using a bicinchoninic acid kit (Thermo Fisher Scientific). Twenty-microgram samples were run on sodium dodecyl sulfate-polyacrylamide gel electrophoresis and transferred to nitrocellulose membrane (Solarbio). The membranes were blocked in 3% bovine serum albumin (Solarbio) for 1 h and then incubated with Abcam primary antibodies overnight and secondary antibody (Abcam, Cambridge, UK) for 2 h. The antibodies included E-cadherin (ab133597, 1:1000 dilution), N-cadherin (ab18203, 1:2000 dilution), Vimentin (ab92547, 1:2000 dilution), CNP (ab183500, 1:1000 dilution), Rac family small GTPase 1 (RAC1) (ab155938, 1:500 dilution), GAPDH (ab37168, 1:3000 dilution), and horseradish peroxidase-labeled IgG (ab97051, 1:5000 dilution). After exposure to enhanced chemiluminescence (Solarbio), the blots were analyzed by Image J, with GAPDH as a normalized reference.

### Dual-luciferase reporter, RNA immunoprecipitation (RIP), and pull-down assays

The wild-type (WT) luciferase reporter vectors (WT-circ-AFF2 and WT-CNP 3′UTR) were constructed by inserting the sequence of circ-AFF2 or CNP 3′UTR containing miR-650 or miR-3612 complementary sites in the pmir-GLO vector (Promega, Madison, WI, USA). The mutant (MUT) luciferase reporter vectors (MUT-circ-AFF2 and MUT-CNP 3′UTR) were constructed by mutating the binding sites of miR-650 or miR-3612. For the dual-luciferase reporter assay, these constructed vectors together with miR-3612 mimic, miR-650 mimic, or miR-NC were transfected in RAFLSs. Luciferase activity was measured with a dual-luciferase analysis kit (Promega) after 24 h post-transfection.

An EZ-Magna RIP kit (Sigma, St. Louis, MO, USA) was utilized for RIP assay. In brief, 1 × 10^7^ RAFLSs were lysed in RIP lysis buffer and incubated with Anti-IgG or Anti-Ago2-coated magnetic beads for 6 h. RNA on the beads was extracted, and enriched circ-AFF2, miR-3612, and miR-650 levels were detected by qRT-PCR as mentioned above.

RNA pull-down assay was performed using a Magnetic RNA Pull-Down Kit (Thermo Fisher Scientific). The biotinylated miR-3612 mimic (Bio-miR-3612), Bio-miR-650, and negative control (Bio-miR-NC) were generated by RiboBio (Guangzhou, China). RAFLSs were transfected with 100 nm biotinylated probes for 48 h and then were lysed using lysis buffer. The lysates were incubated with streptavidin-coated magnetic beads overnight. Then RNA enriched on the beads was isolated, and circ-AFF2 enrichment level was measured by qRT-PCR.

### Statistical analysis

Three independent experiments were performed. Data were shown as mean ± standard deviation (SD). The comparison between groups was conducted by the Student *t*-test or one-way analysis of variance followed via Tukey’s post hoc test as appropriate, processed using GraphPad Prism 8 (GraphPad Inc., La Jolla, CA, USA). The difference was statistically significant at *P* < 0.05.

## Results

### Circ-AFF2 level is increased in RA

**Fig. 1:**
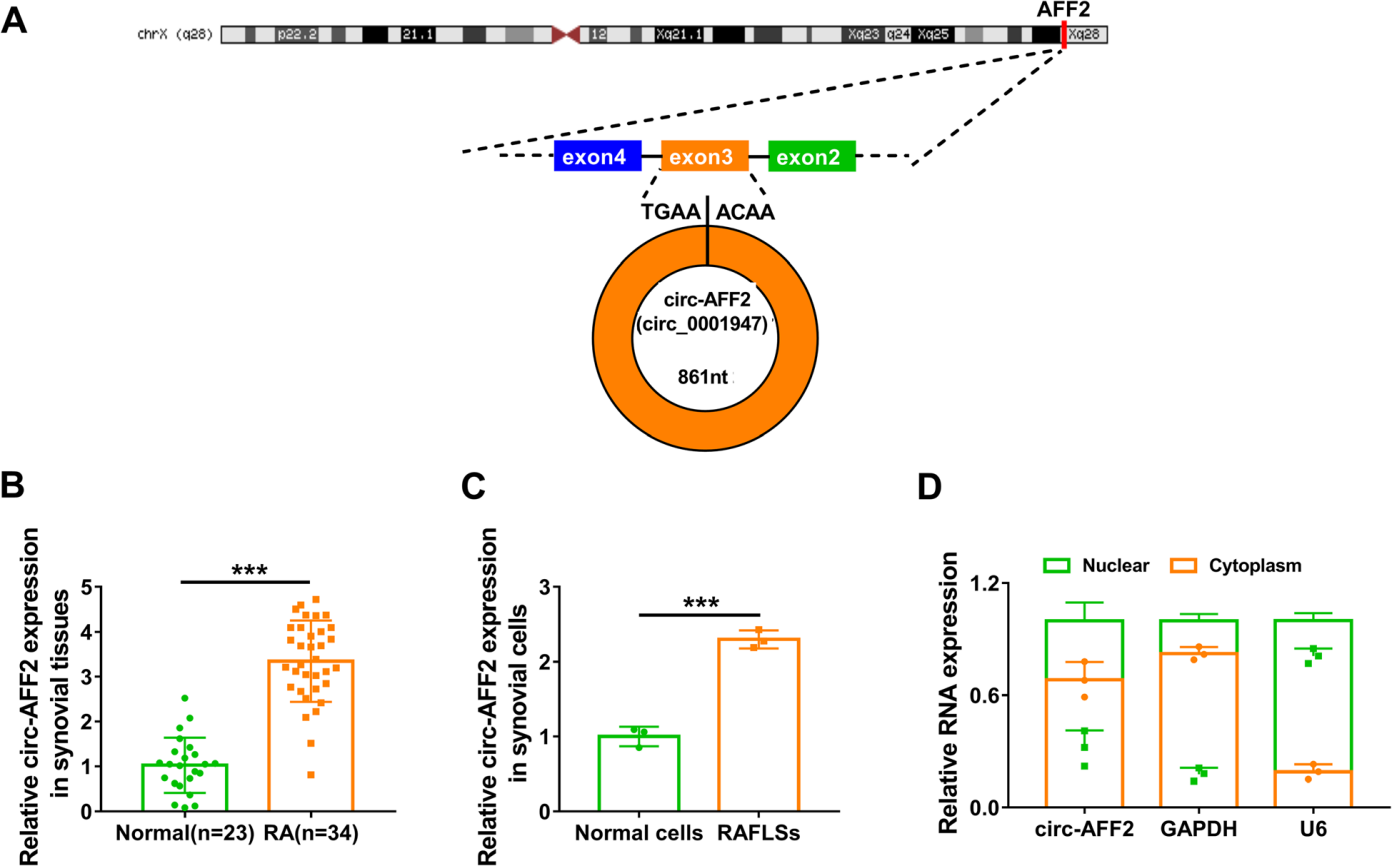
Circ-AFF2 expression in RA. **a** The information on circ-AFF2. **b** Circ-AFF2 level was measured via qRT-PCR in synovial tissues from RA or normal subjects. **c** Circ-AFF2 expression was detected by qRT-PCR in RAFLSs and normal cells. **d** Circ-AFF2, GAPDH, and U6 levels were examined using qRT-PCR in nuclear and cytoplasm of RAFLSs. ***P < 0.001

Circ-AFF2 (hsa_circ_0001947) was studied by CircView, which showed that circ-AFF2 was back-spliced by the exon 3 of AFF2 transcripts (Fig. 1a). To explore whether circ-AFF2 was related to RA development, circ-AFF2 expression change was analyzed in synovial tissues from RA (*n* = 34) or normal subjects (*n* = 23). Compared with normal subjects, circ-AFF2 level was markedly enhanced in RA synovial samples (Fig. 1b). Furthermore, the fibroblast-like synoviocytes were isolated from RA tissues (RAFLSs) or normal subjects (normal cells). Circ-AFF2 expression was higher in RAFLSs than in normal cells (Fig. 1c). Additionally, circ-AFF2 expression distribution was analyzed by using GAPDH and U6 as a cytoplasm or nucleus reference. Results showed circ-AFF2 was mostly located in the cytoplasm of RAFLSs (Fig. 1d). These results suggested circ-AFF2 might have important roles in RA development.

### Circ-AFF2 promotes cell proliferation, inflammatory response, migration, and invasion

**Fig. 2:**
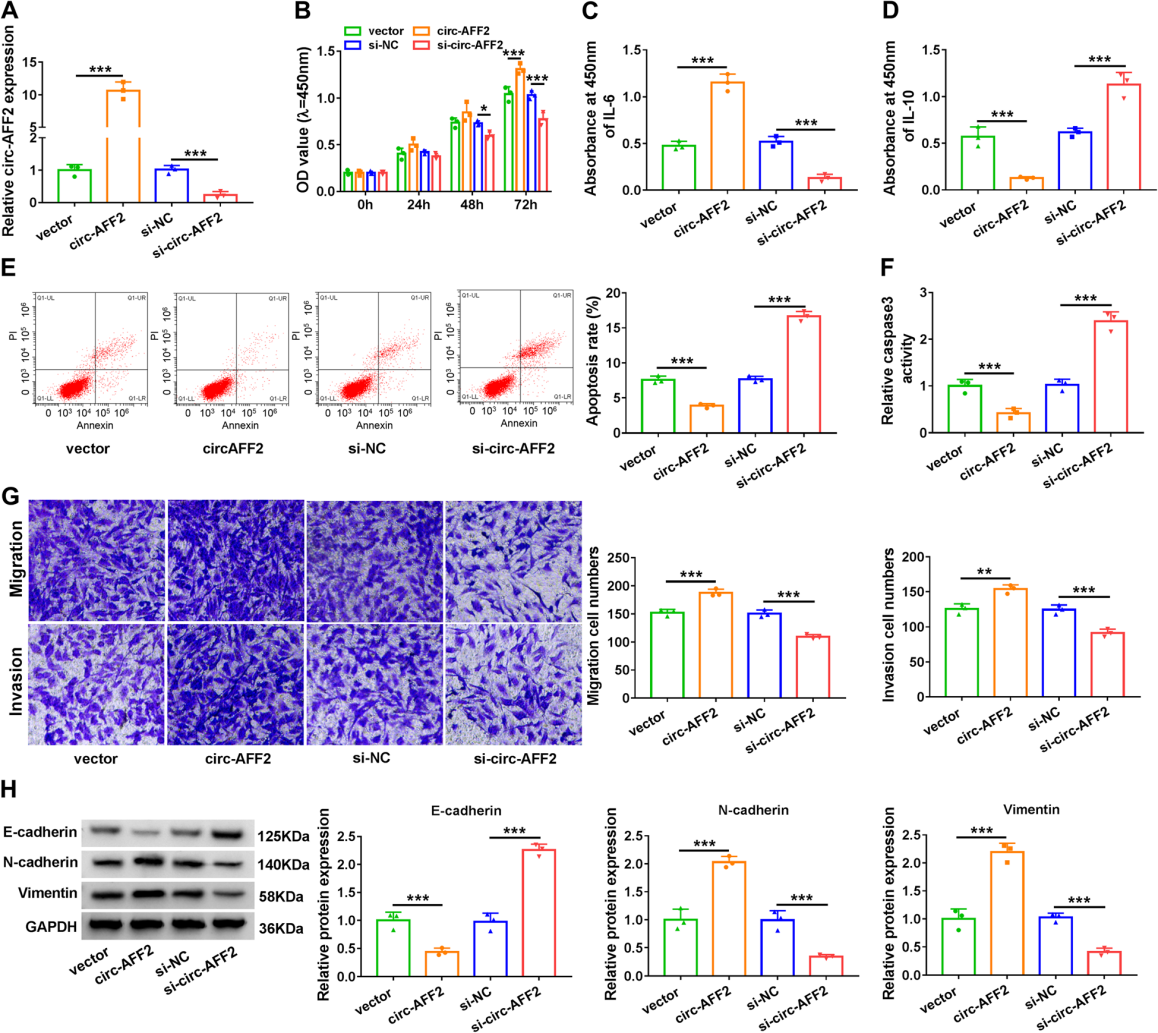
The effect of circ-AFF2 on cell proliferation, inflammatory response, apoptosis, migration, invasion, and EMT. RAFLSs were transfected with vector, circ-AFF2 overexpression vector, si-NC, or si-circ-AFF2. **a** Circ-AFF2 expression was measured via qRT-PCR in the transfected cells. **b** Cell proliferation was detected by CCK-8 in the transfected cells. **c**, **d** IL-6 and IL-10 levels were determined with ELISA in the transfected cells. **e**, **f** Cell apoptosis was examined via flow cytometry and caspase3 activity. **g** Cell migration and invasion were detected using Transwell analysis in cells. **h** E-cadherin, N-cadherin, and Vimentin levels were determined utilizing Western blotting. *P < 0.05, **P < 0.01, ***P < 0.001

To study the role of circ-AFF2 in RAFLS processes, circ-AFF2 was overexpressed or knocked down in RAFLSs. Circ-AFF2 expression level in RAFLSs was increased 11.94-fold by transfection of circ-AFF2 overexpression vector, and decreased 80% by addition of si-circ-AFF2 (Fig. 2a). RAFLS proliferation was significantly promoted by circ-AFF2 overexpression, but decreased by circ-AFF2 knockdown (Fig. 2b). Moreover, circ-AFF2 overexpression evidently increased secretion of IL-6 and decreased IL-10 level in RAFLSs, while silencing circ-AFF2 led to an opposite effect (Fig. 2c, d). Additionally, circ-AFF2 upregulation clearly reduced cell apoptosis and caspase3 activity in RAFLSs, but circ-AFF2 knockdown caused an opposite effect (Fig. 2e, f). Furthermore, circ-AFF2 addition markedly enhanced migration and invasion of RAFLSs, but silencing circ-AFF2 inhibited the migratory and invasive abilities (Fig. 2g). In addition, epithelial-mesenchymal transition (EMT) was associated with cell migration and invasion, and related protein level was detected in RAFLSs. Results showed that circ-AFF2 overexpression notably resulted in reduction of E-cadherin and elevation of N-cadherin and Vimentin, while circ-AFF2 knockdown played opposite effects on these protein levels (Fig. 2h). These data indicated circ-AFF2 might promote RAFLS proliferation, inflammatory response, migration, and invasion.

### MiR-650 is targeted by circ-AFF2

**Fig. 3:**
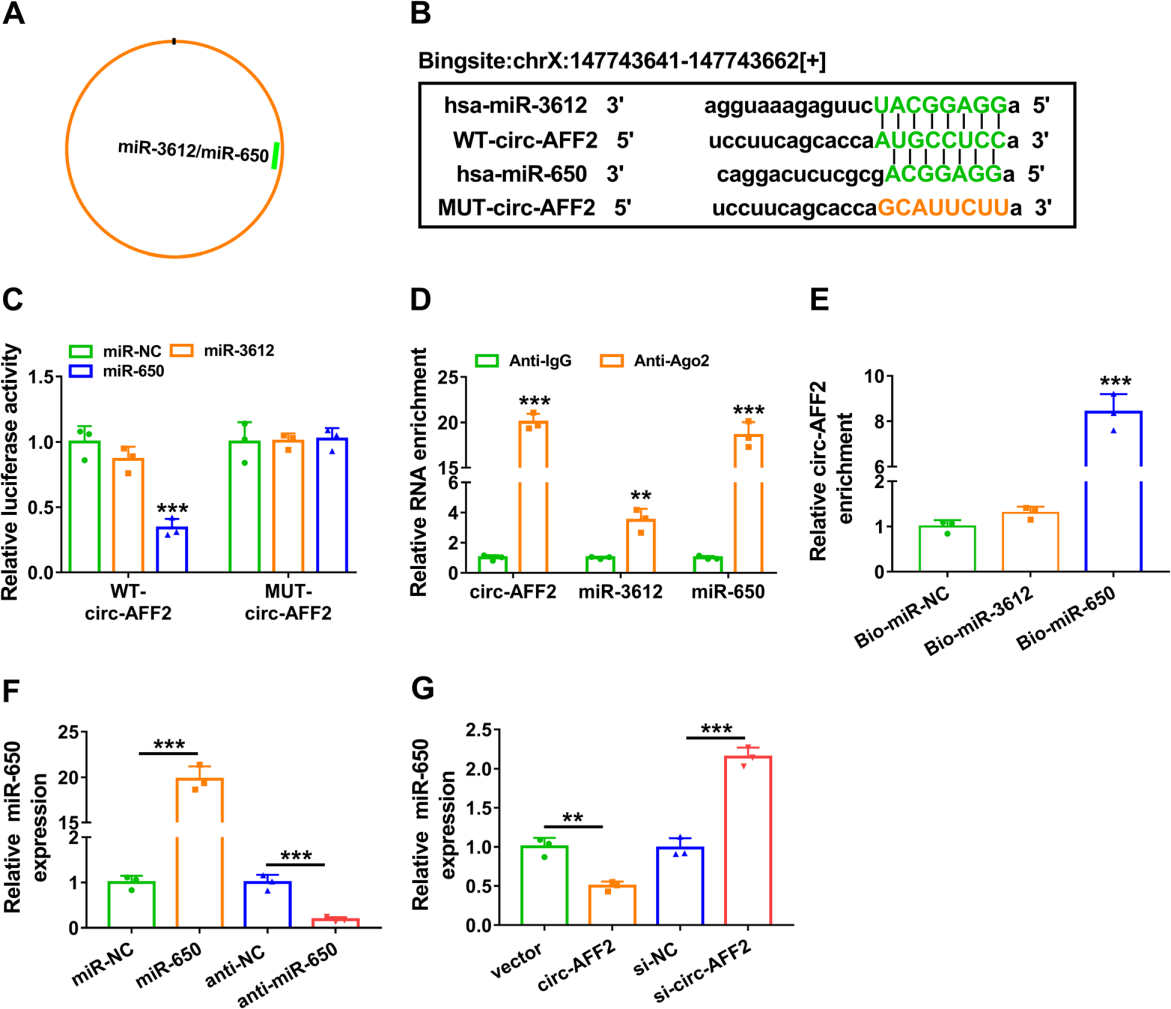
The target relationship of circ-AFF2 on miR-650. **a** The targets of circ-AFF2 were predicted by starBase. **b** The binding sites of circ-AFF2 and miR-3612 or miR-650. **c** Luciferase activity was detected in cells transfected with WT-circ-AFF2 or MUT-circ-AFF2 and miR-NC, miR-3612 mimic, or miR-650 mimic. **d** Circ-AFF2, miR-3612, and miR-650 levels were determined by qRT-PCR after Ago2 or IgG RIP assay. **e** Circ-AFF2 expression was measured with qRT-PCR after RNA pull-down assay using Bio-miR-NC, Bio-miR-3612, or Bio-miR-650. **f** MiR-650 expression was detected by qRT-PCR in RAFLSs transfected with miR-NC, miR-650 mimic, anti-NC, or anti-miR-650. **g** MiR-650 level was measured using qRT-PCR in RAFLSs with transfection of vector, circ-AFF2 overexpression vector, si-NC, or si-circ-AFF2. **P < 0.01, ***P < 0.001

To explore the regulatory network addressed by circ-AFF2, we predicted the targets of circ-AFF2 (hsa_circ_0001947) by starBase. Two miRNAs (miR-3612 and miR-650) were predicted as the potential targets of circ-AFF2 (Fig. 3a). Predicted binding sequences are displayed in Fig. 3b, and WT-circ-AFF2 and MUT-circ-AFF2 luciferase reporter vectors were constructed. The luciferase activity of WT-circ-AFF2 was obviously decreased by miR-650 mimic, but it was not affected by miR-3612 mimic (Fig. 3c). Moreover, the effect of miR-650 mimic on the luciferase activity was abolished in the MUT-circ-AFF2 group (Fig. 3c). In addition, the RIP assay showed circ-AFF2, miR-3612, and miR-650 might be enriched in an Ago2-dependent manner (Fig. 3d). And RNA pull-down analysis displayed circ-AFF2 could bind with miR-650, but not with miR-3612 (Fig. 3e). Furthermore, the overexpression or knockdown efficacy of miR-650 mimic or inhibitor (anti-miR-650) is confirmed in Fig. 3f. Additionally, the influence of circ-AFF2 on miR-650 expression was investigated in RAFLSs. Results showed circ-AFF2 overexpression evidently reduced miR-650 level, while circ-AFF2 knockdown enhanced miR-650 expression (Fig. 3g). These results suggested circ-AFF2 could target miR-650.

### MiR-650 knockdown attenuates the effect of silencing circ-AFF2 on RAFLS proliferation, inflammatory response, migration, and invasion

**Fig. 4:**
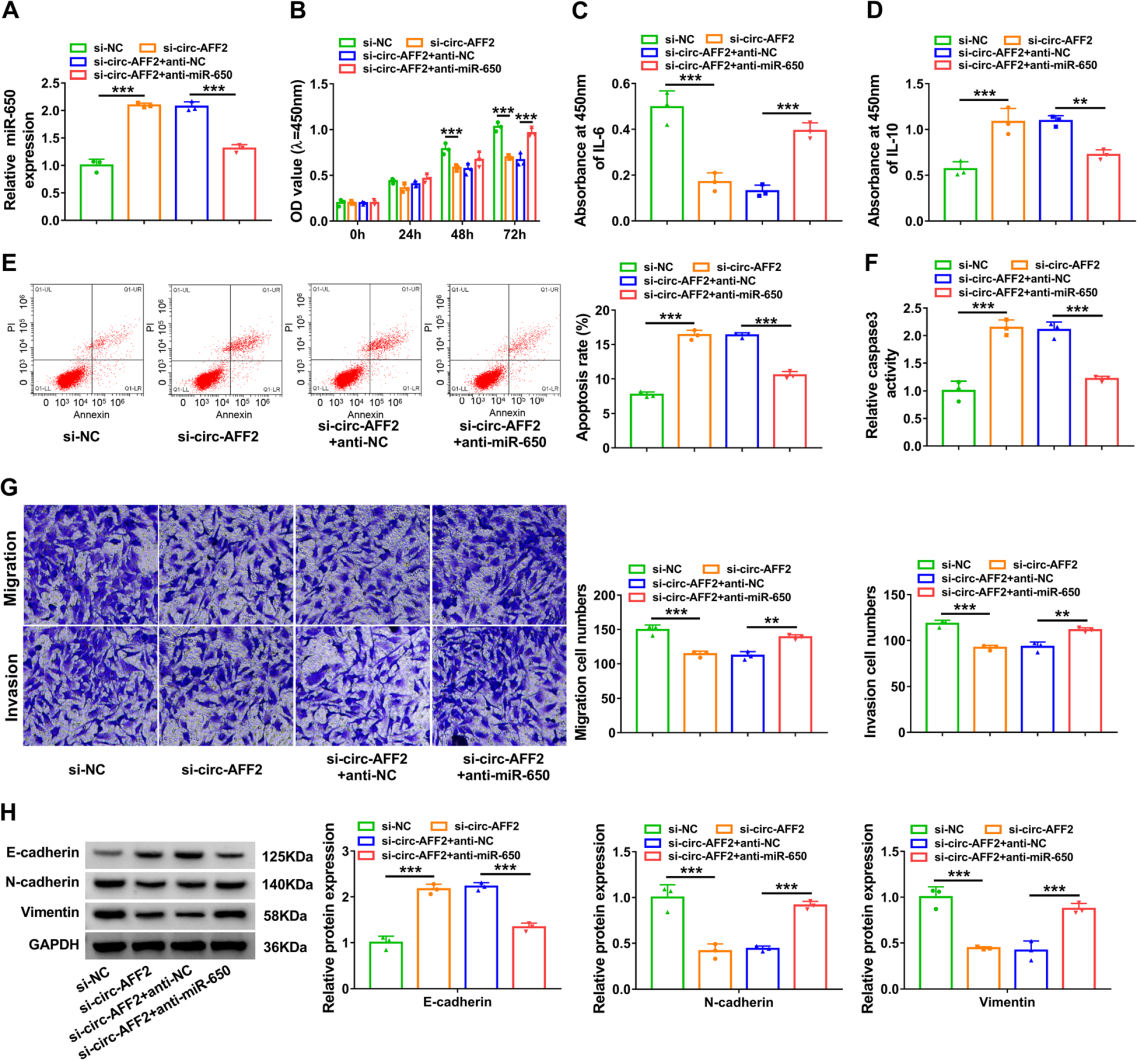
The effect of miR-650 knockdown on silencing circ-AFF2-mediated cell proliferation, inflammatory response, apoptosis, migration, invasion, and EMT. RAFLSs were transfected with si-NC, si-circ-AFF2, and si-circ-AFF2 + anti-NC or anti-miR-650. **a** MiR-650 level was determined by qRT-PCR in the transfected cells. **b** Cell proliferation was measured using CCK-8 in the transfected cells. **c**, **d** IL-6 and IL-10 levels were examined via ELISA. **e**, **f** Cell apoptosis was assessed with flow cytometry and caspase3 activity. **g** Cell migration and invasion were measured through Transwell analysis. **h** E-cadherin, N-cadherin, and Vimentin levels were determined using Western blotting. **P < 0.01, ***P < 0.001

To probe if miR-650 was related to circ-AFF2-mediated cell processes, RAFLSs were transfected with si-NC, si-circ-AFF2, and si-circ-AFF2 + anti-NC or anti-miR-650. The transfection of anti-miR-650 weakened silencing circ-AFF2-induced miR-650 upregulation (Fig. 4a). MiR-650 knockdown rescued interference of circ-AFF2-mediated proliferation reduction (Fig. 4b). In addition, miR-650 downregulation reversed knockdown of circ-AFF2-mediated inhibition of IL-6 secretion and increase of IL-10 (Fig. 4c, d). Furthermore, miR-650 knockdown weakened interference of circ-AFF2-induced promotion of apoptosis and caspase3 activity (Fig. 4e, f). Additionally, miR-650 downregulation attenuated knockdown of circ-AFF2-mediated inhibition of cell migration and invasion (Fig. 4g). Moreover, miR-650 knockdown reversed the elevation of E-cadherin and the decrease of N-cadherin and Vimentin mediated by silencing circ-AFF2 (Fig. 4h). These results indicated circ-AFF2 modulated RAFLS processes by regulating miR-650.

### CNP is targeted by miR-650 and regulated via the circ-AFF2/miR-650 axis

**Fig. 5:**
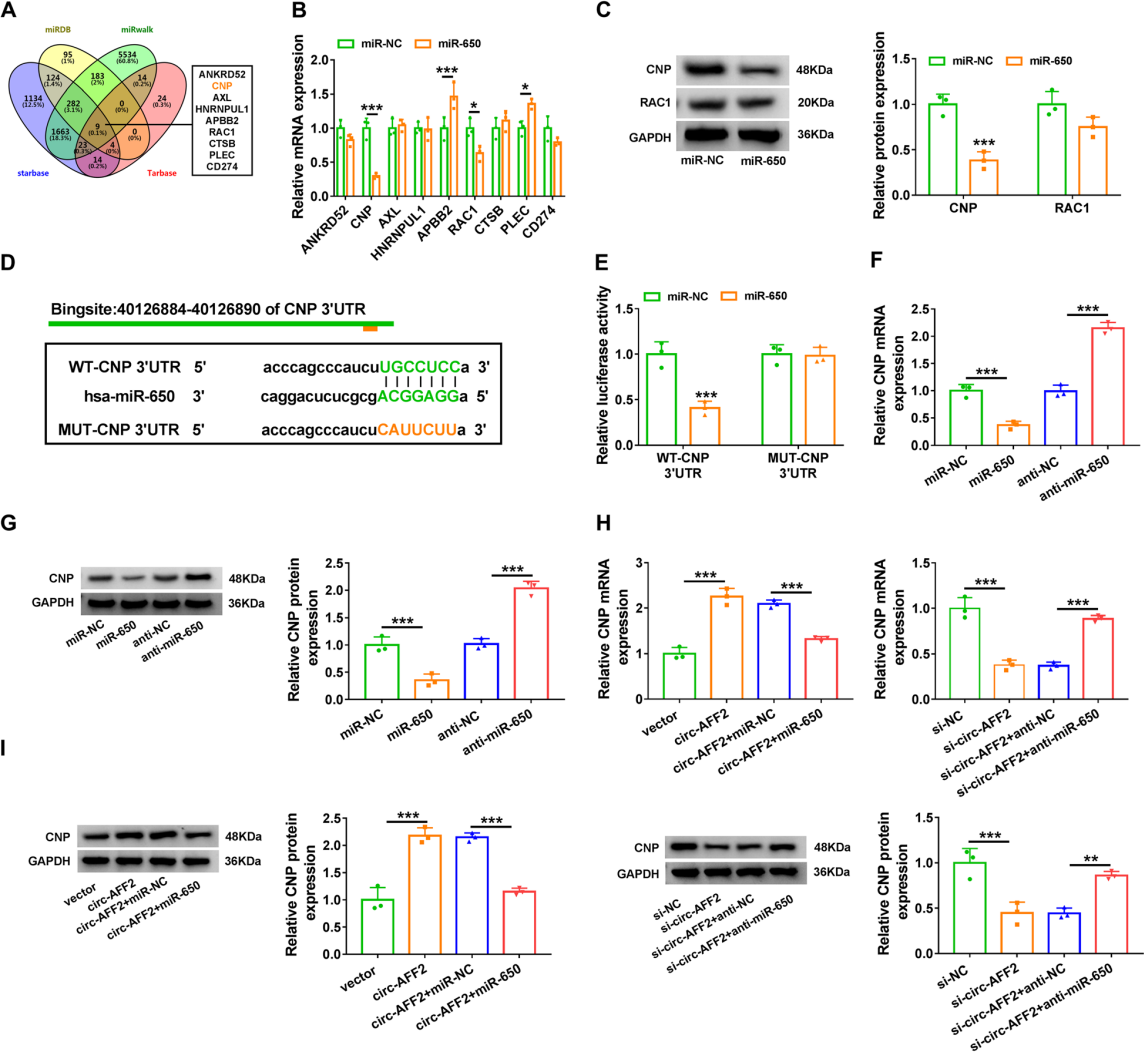
The target association of miR-650 and CNP. **a** The targets of miR-650 were predicted by miRDB, miRwalk, starBase, and Tarbase. **b** The levels of the predicted targets were detected by qRT-PCR in cells transfected with miR-NC or miR-650 mimic. **c** CNP and RAC1 protein levels were detected via Western blotting in cells transfected with miR-NC or miR-650 mimic. **d** The target sites of miR-650 and CNP. **e** Luciferase activity was measured in cells transfected with WT-CNP 3′UTR or MUT-CNP 3′UTR and miR-NC or miR-650 mimic. **f**, **g** CNP level was measured via qRT-PCR and Western blotting in RAFLSs transfected with miR-NC, miR-650 mimic, anti-NC, or anti-miR-650. **h**, **i** CNP level was determined by qRT-PCR and Western blotting in RAFLSs with transfection of vector, circ-AFF2 overexpression vector, circ-AFF2 overexpression vector + miR-NC or miR-650 mimic, si-NC, si-circ-AFF2, and si-circ-AFF2 + anti-NC or anti-miR-650. *P < 0.05, ** *P*< 0.01, *** *P*< 0.001

To further explore the regulatory network, the targets of miR-650 were analyzed by miRDB, miRwalk, starBase, and Tarbase. There were 9 overlapping targets predicted by the four tools online (Fig. 5a). The effect of miR-650 on their expression levels was investigated in RAFLSs. As shown in Fig. 5b, c, CNP level was reduced most by miR-650 overexpression. Hence, CNP was selected as a potential target for further experiments. Predicted binding sites between miR-650 and CNP are displayed in Fig. 5d. To validate this prediction, WT-CNP 3′UTR and MUT-CNP 3′UTR luciferase reporter vectors were constructed. The luciferase activity of WT-CNP 3′UTR was clearly reduced by miR-650 mimic, while the activity of MUT-CNP 3′UTR was not affected via miR-650 (Fig. 5e). Moreover, CNP expression was negatively regulated by miR-650 (Fig. 5f, g). In addition, circ-AFF2 overexpression or downregulation obviously increased or decreased CNP expression in RAFLSs, and this effect was reversed by miR-650 mimic or anti-miR-650 (Fig. 5h, i). These data suggested circ-AFF2 could target CNP through miR-650.

### MiR-650 constrains cell proliferation, inflammatory response, migration, and invasion by decreasing CNP

**Fig. 6:**
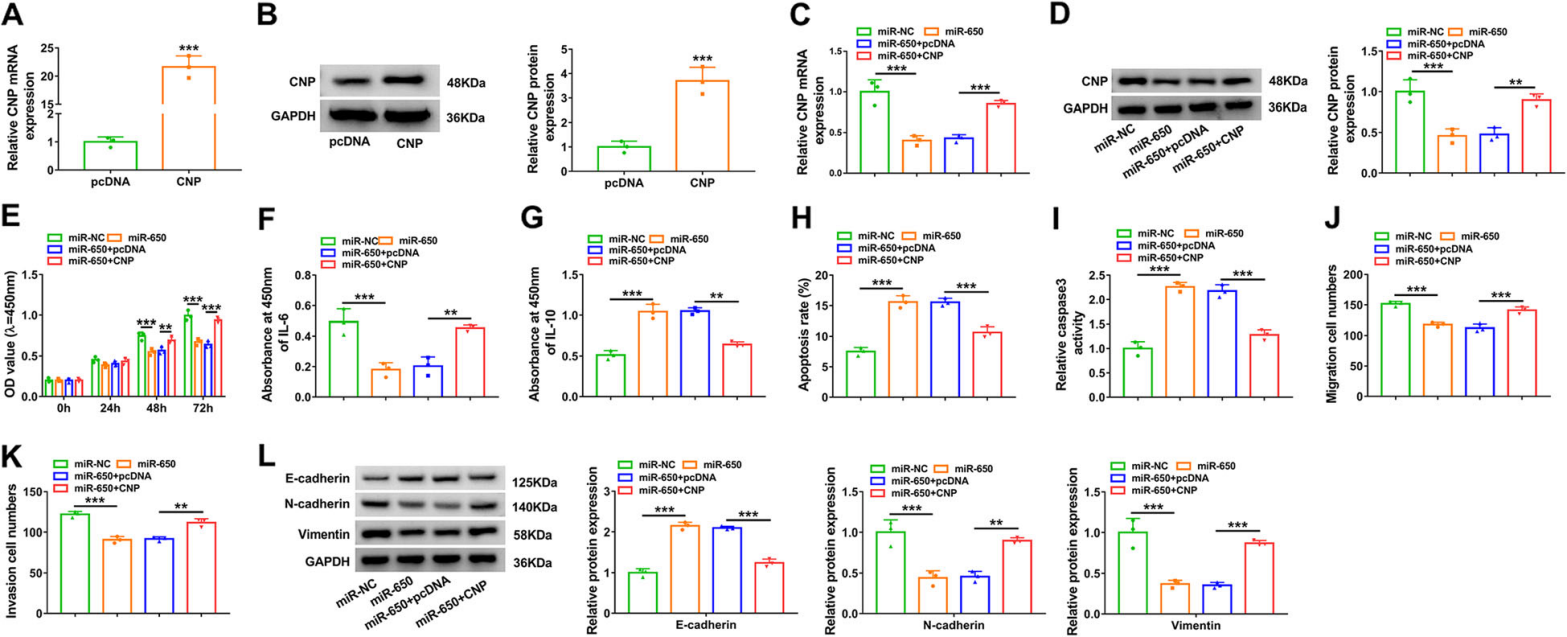
The effect of miR-650 and CNP overexpression on cell proliferation, inflammatory response, apoptosis, migration, invasion, and EMT. **a**, **b** CNP abundance was examined via qRT-PCR and Western blotting in RAFLSs transfected with pcDNA or CNP overexpression vector. CNP expression (**c**, **d**), cell proliferation (**e**), IL-6 and IL-10 levels (**f**, **g**), apoptosis (**h**), caspase3 activity (**i**), migration (**j**), invasion (**k**), and expression of E-cadherin, N-cadherin, and Vimentin (**l**) were determined in RAFLSs transfected with miR-NC, miR-650 mimic, and miR-650 mimic + pcDNA or CNP overexpression vector by qRT-PCR, Western blotting, CCK-8, and ELISA or Transwell assay, respectively. **P < 0.01, ***P < 0.001

To explore the function of the miR-650/CNP axis in RAFLS processes, cells were transfected with miR-NC, miR-650 mimic, and miR-650 mimic + pcDNA or CNP overexpression vector. The transfection of CNP overexpression vector effectively enhanced CNP expression in RAFLSs (Fig. 6a, b). Moreover, addition of CNP overexpression vector restored CNP level that was decreased by miR-650 mimic (Fig. 6c, d). CCK-8 assay showed miR-650 mimic evidently reduced RAFLS proliferation, and CNP upregulation reversed this effect (Fig. 6e). Additionally, miR-650 overexpression significantly constrained IL-6 expression and increased IL-10 secretion in RAFLSs, and these events were reversed by CNP overexpression (Fig. 6f, g). Furthermore, miR-650 overexpression clearly caused apoptosis promotion and increased caspase3 activity, and this effect was weakened via CNP upregulation (Fig. 6h, i). In addition, miR-650 markedly restrained cell migration, invasion, and EMT, which was mitigated by CNP addition (Fig. 6j–l). These results indicated miR-650 inhibited RAFLS processes by regulating CNP.

## Discussion

RA is a joint disease characterized by the chronic inflammation of the synovial membrane, and significant improvement is involved in the diagnosis and therapy of RA [24]. However, approximately 20–25% of patients do not reach the low disease activity [24]. Fibroblast-like synoviocytes are involved in the overproduction of enzymes and cytokines that are associated with cartilage degradation and immune cell infiltration [25]. CircRNAs are involved in various diseases including RA, but their roles have not been broadly explored [26]. Here we explored the role of circ-AFF2 in RA and found the promoting function of circ-AFF2 on RAFLS processes. Moreover, we confirmed it was associated with the miR-650/CNP axis.

Our study showed upregulated circ-AFF2 in RA synovial tissues and RAFLSs, which was also consistent with that in the peripheral blood of RA patients [11]. This finding suggested RA development and RAFLS processes might be related to the dysregulated circ-AFF2. Targeting RAFLS apoptosis is an important strategy for RA treatment [27]. Here we found silencing circ-AFF2 attenuated cell proliferation and promoted apoptosis. Furthermore, the balance of pro-inflammatory IL-6 and anti-inflammatory IL-10 regulates the inflammatory response in RA [28–30]. By detecting their secretion levels, we confirmed circ-AFF2 knockdown mitigated the inflammatory response in RAFLSs by decreasing IL-6 and increasing IL-10. Additionally, the migration, invasion, and gain of mesenchymal characteristics of RAFLSs are also key events in RA [31–33]. By measuring cell migration, invasion, and EMT-related protein expression [34], we found that circ-AFF2 knockdown repressed RAFLS migration and invasion. Moreover, circ-AFF2 overexpression promoted RAFLS proliferation, inflammatory response, migration, and invasion. Collectively, circ-AFF2 might serve as an important intervention for RA therapy.

Moreover, the regulatory network mediated by circ-AFF2 was explored. We firstly confirmed circ-AFF2 could sponge miR-650. Previous studies reported miR-650 promoted cell proliferation, migration, and invasion in oral cancer and hepatocellular carcinoma [35, 36], while miR-650 suppressed cell proliferation in glioma and acute myeloid leukemia [37, 38]. More importantly, a former work indicated miR-650 suppressed proliferation, migration, and invasion of RAFLSs by modulating protein kinase B2 (AKT2) [16]. Similarly, we also found this function of miR-650 in our study. Moreover, we confirmed miR-650 was modulated by circ-AFF2 to take part in the regulation of RAFLS processes. Additionally, we further confirmed CNP was a downstream target of miR-650. Multiple evidences suggested CNP had a pro-proliferation role in various cell lines, like melanoma cells, mouse Leydig cells, and osteoblastic cells [39–41]. However, the function of CNP on RAFLS processes remains unclear. Our study found CNP upregulation attenuated the suppressive role of miR-650 in RAFLS proliferation, migration, and invasion. In addition, we firstly confirmed circ-AFF2 regulated CNP expression indirectly through miR-650. In this way, this axis promoted RAFLS proliferation, inflammatory response, migration, and invasion, thereby contributing to RA development.

Our study performed three independent experiments using the primary RAFLSs, which might represent the physiology of RA patients. Moreover, we firstly confirmed the involvement of the circ-AFF2/miR-650/CNP network in RAFLS dysfunction, indicating the importance and clinical expectation of this axis in RA progression and treatment. However, there were some limitations in the current study. First, we measured circ-AFF2 expression in RA patients (*n* = 34), and increased sample sizes are needed in a further study. Second, the downstream pathway mediated by the circ-AFF2/miR-650/CNP network should be explored. Third, our conclusions were based on the in vitro experiments, and animal studies are needed in the future.

## Conclusion

Circ-AFF2 upregulation facilitated proliferation, inflammatory response, migration, and invasion of RAFLSs, partly by the miR-650/CNP axis. This study provided a new regulatory network of circ-AFF2/miR-650/CNP in RAFLSs. We expect this work will serve as a valuable resource in the future clinical treatment of RA.

## Data Availability

Not applicable

## Abbreviations

RA: Rheumatoid arthritis
RAFLSs: Rheumatoid arthritis fibroblast-like synoviocytes
CNP: Cyclic nucleotide 3′-phosphodiesterase
EMT: Epithelial-mesenchymal transition
ELISA: Enzyme-linked immunosorbent assay
FITC: Fluorescein isothiocyanate
